# Consideration of Cosmetic Surgery As Part of Women’s Benefit-Provisioning Mate Retention Strategy

**DOI:** 10.3389/fpsyg.2017.01389

**Published:** 2017-08-14

**Authors:** Mohammad Atari, Nicole Barbaro, Yael Sela, Todd K. Shackelford, Razieh Chegeni

**Affiliations:** ^1^Department of Psychology, University of Tehran Tehran, Iran; ^2^Department of Psychology, Oakland University, Rochester MI, United States; ^3^Department of Psychology, Alzahra University Tehran, Iran

**Keywords:** mate retention, cosmetic surgery, physical attractiveness, sex differences, evolutionary psychology

## Abstract

Individuals perform mate retention behaviors to minimize the risk of partner infidelity and relationship dissolution. The current study investigates whether consideration of cosmetic surgery can be conceptualized as part of a broader strategy of mate retention for women, but not men. We hypothesized that women’s consideration of cosmetic surgery would be positively associated with performance frequencies of Benefit-Provisioning and Cost-Inflicting mate retention behaviors. We recruited 203 individuals (54% women) in committed heterosexual relationships from Tehran, Iran. Results indicate a positive association between consideration of cosmetic surgery and Benefit-Provisioning mate retention behaviors for women, but not men. There was no association between consideration of cosmetic surgery and Cost-Inflicting mate retention behaviors. Women therefore may consider cosmetic surgery to improve their physical attractiveness as part of a Benefit-Provisioning strategy to retain a long-term mate. We discuss limitations of the study and highlight future directions for research from an evolutionary perspective.

## Introduction

Partner infidelity is a considerable threat to romantic relationships, and is associated with a greater likelihood of relationship dissolution (Allen and Atkins, 2012). Preventing partner infidelity in long-term relationships has been an adaptive problem throughout human evolutionary history (Buss, 1988). A man whose partner commits sexual infidelity is at risk for cuckoldry—investing resources into genetically unrelated offspring. A woman whose partner commits emotional infidelity is at risk for losing partner-provisioned resources for her and her offspring. Men and women therefore use a variety of strategies to reduce the risk of partner infidelity, and to maintain their long-term romantic relationships (Buss, 1988; Barbaro et al., 2015).

Men and women perform mate retention behaviors to reduce the likelihood of their partner’s infidelity or relationship dissolution (Buss, 1988). Mate retention behaviors can range from socially acceptable behaviors (e.g., appearance enhancement) to socially unacceptable behaviors (e.g., physical violence toward potential rivals). Mate retention behaviors are subsumed by two superordinate domains of Benefit-Provisioning mate retention and Cost-Inflicting mate retention (Miner et al., 2009; Lopes et al., 2016; Atari et al., 2017a). Benefit-Provisioning mate retention includes behaviors that reduce the likelihood of partner infidelity by increasing relationship satisfaction. Cost-Inflicting mate retention includes behaviors that reduce the likelihood of partner infidelity by lowering the partner’s self-esteem (Miner et al., 2009).

Benefit-Provisioning and Cost-Inflicting mate retention behaviors can either be directed toward an individual’s romantic partner (intersexual competition), or toward potential rivals (intrasexual competition) (Buss, 1988). Women, in particular, compete intersexually and intrasexually within the domain of physical attractiveness (Buss and Schmitt, 1993). As part of Benefit-Provisioning mate retention, women (relative to men) report more frequently engaging in Appearance Enhancement behaviors (e.g., making sure they look “extra attractive” for their partner) to increase their partner’s perception of their mate value and therefore their partner’s satisfaction with the relationship. As part of Cost-Inflicting mate retention, on the other hand, women derogate a female rival’s physical attractiveness (e.g., pointing out to their partner the flaws of another woman) to decrease the rival’s mate value, thereby increasing their own relative mate value.

One way a woman can increase her perceived mate value is through the use of cosmetics (Etcoff et al., 2011) and undergoing cosmetic surgery (see Swami et al., 2008). Cosmetic enhancement (e.g., use of cosmetics, body modification) has existed for thousands of years (Kalantar-Hormozi, 2013; Frederick et al., 2016). For example, techniques to reshape or reconstruct nasal deformities were practiced by ancient Greeks, Europeans, and Indians long before advances in modern medical procedures (Frederick et al., 2016). Breast implants—made of paraffin, beeswax, and vegetable oil—were used to enlarge breasts in the 19th and early 20th centuries (Frederick et al., 2016). Cosmetic surgery, therefore, is one method by which an individual can increase their perceived physical attractiveness (Etcoff et al., 2011). Because women’s mate value is more dependent on physical attractiveness than is men’s mate value (e.g., Conroy-Beam et al., 2015), it is not surprising that over 90% of cosmetic surgery patients are women (Frederick et al., 2016). Recent qualitative research suggests that a large number of female patients pursue cosmetic surgery for relationship-related reasons (Locatelli et al., 2017).

Women, more than men, may therefore consider cosmetic surgery as a means to improve their relationship quality, such that cosmetic surgery is a modern means of enhancing physical attractiveness. Women who undergo cosmetic surgery aim to improve their perceived attractiveness. Post-operative assessments indicate that women appear younger, more likeable, and more feminine following cosmetic procedures (Reilly et al., 2015). Men, in contrast, are less interested in cosmetic surgery (Frederick et al., 2007) as a means of improving their physical attractiveness because men’s (relative to women’s) mate value depends upon their social status and resource-acquisition abilities (Buss and Schmitt, 1993).

The current study examines whether women’s, but not men’s, consideration of cosmetic surgery is associated with mate retention behaviors. Interest in cosmetic surgery may be correlated with Benefit-Provisioning mate retention. Women’s cosmetic surgery can augment their physical attractiveness and decrease their perceived age (Reilly et al., 2015). Enhanced physical attractiveness may lead to an increase in relationship satisfaction (see Swami and Mammadova, 2012)—consistent with the conceptualization of Benefit-Provisioning mate retention (Miner et al., 2009). Cosmetic surgery may be associated with Cost-Inflicting mate retention. Because cosmetic surgery artificially enhances a woman’s perceived attractiveness, women who undergo cosmetic procedures may thereby indirectly derogate intrasexual competitors. Women’s enhanced appearance, moreover, may inflate mate value discrepancy within a romantic dyad, consequently resulting in a decrease in their partner’s self-esteem—consistent with the conceptualization of Cost-Inflicting mate retention (Sela et al., 2016). It was therefore expected that consideration of cosmetic surgery will be positively associated with both Benefit-Provisioning mate retention (Hypothesis 1) and Cost-Inflicting (Hypothesis 2) mate retention for women, but not men.

## Materials and Methods

### Participants

A convenience sample of 205 participants in a committed heterosexual relationship was recruited from Tehran, Iran. Data from two participants were excluded from analyses because they did not complete one of the target measures. The final sample consisted of 203 participants (54% women). Participants were between 19 and 61 years (*M* = 32.0, *SD* = 7.9) and the mean relationship length was 78.3 months (*SD* = 78.5). Regarding education, two participants completed some high school, 25 had a high school diploma, 23 had an associate’s degree, 58 had a bachelor’s degree, 56 had a master’s degree, 38 had a doctorate degree, and one participant did not report educational background.

### Procedures

Potential participants were approached by a researcher in public spaces in Tehran, Iran. Participants were eligible to participate if they were at least 18 years of age and in a committed, heterosexual relationship. Interested and eligible participants gave consent. Full ethical review and approval was not required for this study in accordance with the national and institutional guidelines. This study was carried out in accordance with the recommendations of the ethics committee of the Department of Psychology of University of Tehran. Consenting participants were then given a paper-and-pencil questionnaire to provide demographic information (age, sex, relationship length, education), and then reported on their consideration of cosmetic surgery and performance of mate retention behaviors (participants also completed other measures unrelated to the study hypotheses). When complete, participants placed their anonymous questionnaire in an envelope and returned the envelope to the researcher. The researcher answered any questions from participants regarding the study’s aims. Participants did not receive compensation.

### Measures

#### Mate Retention Behaviors

Participants completed the Persian translation of the Mate Retention Inventory-Short Form (MRI-SF; Buss et al., 2008; Atari et al., 2017a). Participants reported how often they performed each of 38 mate retention behaviors on a 4-point Likert-type scale ranging from 0 (*Never*) to 3 (*Often*). Atari et al. (2017a) reported satisfactory psychometric properties of the Persian translation of the MRI-SF in Iran. The Persian translation of the MRI-SF demonstrates a two-component structure, consistent with Miner et al. (2009) and Lopes et al. (2016). Following Atari et al. (2017a), the scores for Benefit-Provisioning mate retention (α = 0.86) and Cost-Inflicting mate retention (α = 0.79) were calculated by averaging participants’ responses to the respective items.

#### Consideration of Cosmetic Surgery

Participants completed the Persian translation of the Acceptance of Cosmetic Surgery Scale (ACSS; Henderson-King and Henderson-King, 2005; Kalantar-Hormozi et al., 2016). We used items from the *Consider* subscale. Participants reported their agreement with five items about considering cosmetic surgery. Participants responded to each item on a 7-point Likert-type scale ranging from 1 (*Strongly disagree*) to 7 (*Strongly agree*). The Persian translation of the ACSS has been used in Iran with adequate reliability and validity (Kalantar-Hormozi et al., 2016). The scores for consideration of cosmetic surgery were calculated by averaging participants’ responses to the five items (α = 0.93).

#### Body Mass Index

Participants reported their weight (Kg) and height (cm). Participants responses were used to calculate body mass index (BMI) by dividing participant’s weight by height-squared (Kg/m^2^). Weight and BMI have been shown to be associated with cosmetic surgery attitudes (Dunaev et al., 2016).

## Results

The associations between considering cosmetic surgery and tactic- and domain-level mate retention behaviors in men and women are separately summarized in **Table 1**. As can be seen in domain-level correlations, women’s interest in cosmetic surgery is significantly associated with Benefit-Provisioning (*r* = 0.23, *p* = 0.01) and Cost-Inflicting (*r* = 0.31, *p* < 0.01) mate retention behaviors. The correlation coefficients between men’s interest in cosmetic surgery and mate retention domains were non-significant (*p*s > 0.34). We conducted Fisher’s *r*-to-*z* transformation on correlations for women (*n* = 110) and men (*n* = 93) to identify sex differences in the magnitude of the correlations coefficients between mate retention behaviors and consideration of cosmetic surgery (see **Table 1**). Zero-order correlations suggested that consideration of cosmetic surgery was negatively associated with education (*r* = -0.28, *p* < 0.01). The correlations between consideration of cosmetic surgery and age (*r* = 0.05, *p* = 0.50), relationship length (*r* = 0.06, *p* = 0.46), and BMI (*r* = -0.07, *p* = 0.34) were non-significant.

**Table 1:** Associations between mate retention variables and considering cosmetic surgery.

Mate retention variables	Considering cosmeticsurgery		Sex differences (*z*)
	Women (*n* = 110)	Men (*n* = 93)	
	
	*r*	*r*	
Vigilance	0.29^∗∗^	0.16	0.96
Concealment of mate	0.14	0.02	0.85
Monopolization of time	0.22^∗^	0.22^∗^	0.01
Jealousy induction	0.05	-0.05	0.70
Punish mate’s infidelity threat	0.24^∗^	0.05	1.36
Emotional manipulation	0.03	0.13	-0.70
Commitment manipulation	0.15	0.14	0.07
Derogation of competitors	0.22^∗^	0.15	0.51
Resource display	0.17	-0.03	1.41
Sexual inducements	0.16	-0.09	1.76
Appearance enhancement	0.14	0.11	0.21
Love and care	0.01	0.13	-0.84
Submission and debasement	0.03	-0.01	0.28
Verbal possession signals	0.14	0.23^∗^	-0.65
Physical possession signals	0.36^∗∗^	-0.04	2.91^∗∗^
Possessive ornamentation	0.22^∗^	-0.04	1.84
Derogation of mate	0.13	0.06	0.49
Intrasexual threats	0.07	-0.13	1.40
Violence against rivals	0.14	0.04	0.71
Benefit-provisioning mate retention	0.23^∗^	0.09	1.01
Cost-inflicting mate retention	0.31^∗∗^	0.10	1.54

*^∗^*p* < 0.05, ^∗∗^*p* < 0.01.*

We conducted a hierarchical moderated regression analysis to test the study hypotheses. In Step 1, the demographic variables of sex, age, education, relationship length, and BMI were entered. In Step 2, Benefit-Provisioning mate retention and Cost-Inflicting mate retention (both variables mean centered) were entered. In Step 3, both two-way interaction terms were entered—Benefit-Provisioning mate retention interacting with sex, and Cost-Inflicting mate retention interacting with sex—with consideration of cosmetic surgery as the dependent variable (see **Table 2**). A two-way interaction emerged between Benefit-Provisioning mate retention and sex.

**Table 2:** Results of moderated regression analyses.

Predictor	B	β	*t*	*R^2^*	Δ*R^2^*
**Step 1**				0.24^∗∗∗^	
Sex (0 = *female*, 1 = *male*)	-1.65	-0.42	-5.54^∗∗∗^		
Age	-0.001	-0.01	-0.06		
Education	-0.33	-0.21	-2.94^∗∗^		
Relationship length	0.00	-0.02	-0.17		
BMI	0.01	0.01	0.18		
**Step 2**				0.30^∗∗∗^	0.06^∗∗^
Sex	-1.91	-0.48	-6.40^∗∗∗^		
Age	0.02	0.06	0.66		
Education	-0.28	-0.18	-2.48^∗^		
Relationship length	0.00	-0.01	-0.15		
BMI	-0.002	-0.003	-0.05		
Benefit-provisioning	0.93	0.23	3.03^∗∗^		
Cost-inflicting	0.34	0.06	0.75		
**Step 3**				0.32^∗∗∗^	0.03
Sex	-1.91	-0.48	-6.47^∗∗∗^		
Age	0.03	0.10	1.08		
Education	-0.27	-0.18	-2.46^∗^		
Relationship length	-0.001	-0.03	-0.38		
BMI	0.002	0.004	0.06		
Benefit-provisioning	1.66	0.40	3.51^∗∗^		
Cost-inflicting	0.23	0.04	0.32		
Benefit-provisioning × sex	-1.36	-0.23	-2.19^∗^		
Cost-inflicting × sex	-0.02	-0.002	-0.02		

*^∗^*p* < 0.05, ^∗∗^*p* < 0.01, ^∗∗∗^*p* < 0.001.*

**FIGURE 1:**
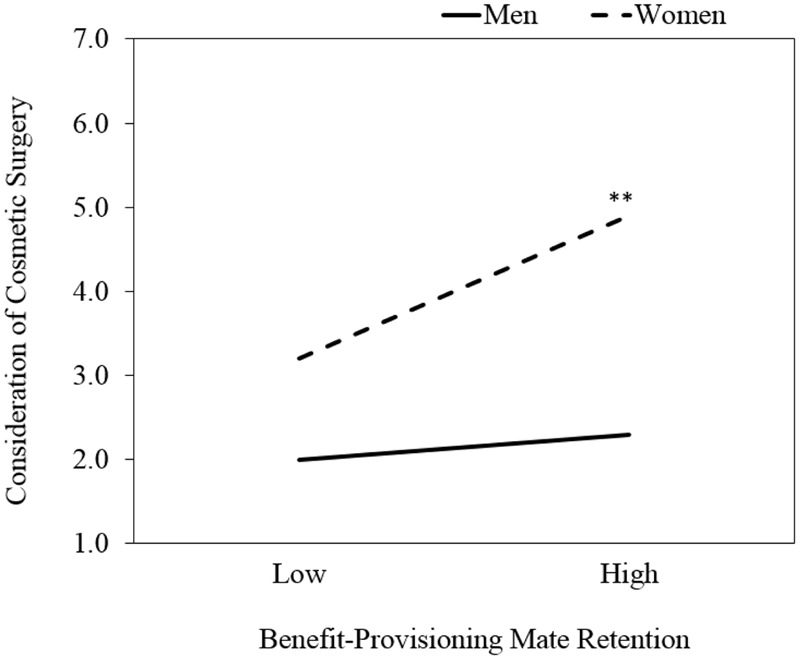
Simple slopes plots for the two-way interaction between Benefit-Provisioning mate retention and sex, predicting consideration of cosmetic surgery. ^∗∗^*p* < 0.01.

We conducted simple slopes analyses, as recommended by Aiken and West (1991), to deconstruct the two-way interaction. The simple slopes tests were conducted using values one standard deviation above the mean to represent individuals who perform relatively more frequent Benefit-Provisioning mate retention, and one standard deviation below the mean to represent individuals who perform relatively less frequent Benefit-Provisioning mate retention. Supporting Hypothesis 1, the slope of the line representing the association between Benefit-Provisioning mate retention and consideration of cosmetic surgery was significant and positive for women (β = 0.35, *t* = 2.77, *p* < 0.01), but not for men (β = 0.09, *t* = 0.81, *p =* 0.42). The predicted values for this interaction are displayed in **Figure 1**. A two-way interaction between Cost-Inflicting mate retention and sex did not emerge and, therefore, Hypothesis 2 was not supported.

## Discussion

The current study examined whether women’s, but not men’s, consideration of cosmetic surgery is associated with Benefit-Provisioning and Cost-Inflicting mate retention. The results revealed a two-way interaction between Benefit-Provisioning mate retention and sex. Hypothesis 1 was supported: for women (but not men), there was a positive association between Benefit-Provisioning mate retention and consideration of cosmetic surgery, such that women who more frequently perform Benefit-Provisioning mate retention also reported greater consideration of cosmetic surgery. Hypothesis 2 was not supported: there was no interaction between Cost-Inflicting mate retention and sex to predict consideration of cosmetic surgery.

In tactic-level analyses, results suggested that women’s consideration of cosmetic surgery is associated with six mate retention tactics (i.e., vigilance, monopolization of time, punish mate’s infidelity threat, derogation of competitors, physical possession signals, and possessive ornamentation). However, men’s consideration of cosmetic surgery was associated with two mate retention tactics (i.e., monopolization of time and verbal possession signals). These findings indicate that women more generally consider cosmetic surgery as a means of retaining their long-term mates by improving their physical attractiveness.

The sex difference of the association between Benefit-Provisioning mate retention and consideration of cosmetic surgery may reflect differences in domains of intersexual competition between men and women. Men, more than women, prioritize physical attractiveness in mates (Buss and Schmitt, 1993). Women who improve their physical attractiveness via cosmetic surgery, therefore, may be able to attract and retain men of higher mate value. For example, women can reconstruct their waist-to-hip ratio—an important indicator of fecundity—to aesthetically ideal proportions by a buttock implant surgery or abdominoplasty. Breast symmetry and size can be augmented or reduced to meet an aesthetic ideal. Men’s attractiveness indicators (e.g., muscularity), however, are less easily modified through cosmetic surgery procedures (Frederick et al., 2016). Men’s social status and resource-acquisition, in contrast to physical attractiveness, moreover, are prioritized more in women’s mate selection (Conroy-Beam et al., 2015). The results of the current study are in accord with the findings of Atari et al. (2017b) who documented that single women who report greater interest in cosmetic surgery set higher standards for potential mates.

Research from an evolutionary psychological perspective has investigated the ways in which aspects of women’s physical attractiveness provide cues to health, youth, and fertility (e.g., Lewis et al., 2015). Men have evolved preferences to desire physically attractive women (Conroy-Beam et al., 2015), and women have historically used a variety of techniques to enhance their attractiveness (Buss, 1988). Cosmetic surgery—as a means to increase perceived attractiveness—may therefore be understood as a modern form of appearance enhancement. The primary aim of cosmetic surgery from an evolutionary psychological perspective may be a modern means of Appearance Enhancement—which women (relative to men) are more likely to deploy as part of a broader strategy of Benefit-Provisioning mate retention (Buss, 1988).

The current study also adds to the growing literature on the psychology of cosmetic surgery (e.g., Kalantar-Hormozi et al., 2016; also see Sarwer et al., 1998). Chegeni and Atari (2017), for example, documented that Machiavellianism is associated with considering cosmetic surgery in women, but not men. Paired with the current findings and previous reports on sex differences in cosmetic surgery settings (Naraghi and Atari, 2016), it may be concluded that the motivations underlying cosmetic surgery differ between the sexes. Men and women may obtain different benefits through cosmetic surgery. Women, for example, may be more successful in attracting and retaining higher quality mates as a result of cosmetic procedures. Our correlational findings further suggests that interest in cosmetic surgery is associated with a set of mate retention tactics in women.

### Limitations and Future Directions

Our results should be interpreted with respect to the limitations of the current research. A general questionnaire was used to assess participant’s interest in cosmetic surgery. Research suggests that procedure-specific questionnaires may provide a more accurate understanding of motivations underlying cosmetic procedures (Naraghi and Atari, 2017). For example, the psychological factors motivating rhinoplasty (i.e., aesthetic nasal surgery) and labiaplasty (i.e., aesthetic labial surgery) might differ given that labiaplasty may be more closely associated with women’s sexual functioning and satisfaction within romantic relationships. It is recommended that future research uses procedure-specific measures to assess interest in cosmetic procedures.

It is important to note that the current study used a non-clinical sample to test the association between consideration of cosmetic surgery and performance frequencies of mate retention behavior. Although the measure of cosmetic surgery used in the current study has high predictive validity (Henderson-King and Henderson-King, 2005), individuals who report an interest in cosmetic surgery may not actually undergo cosmetic surgery. Future research therefore may benefit from investigating associations between mate value and mate retention behaviors, for example, in a clinical sample of men and women who have undergone cosmetic procedures. Such evolutionarily-informed research will generate useful information for medical and mental health communities and the patients themselves.

The current study did not investigate the potential role of personality and individual difference traits in moderating the association between mate retention behavior and consideration of cosmetic surgery. For example, the normative personality dimensions are associated with performance frequencies of mate retention behaviors (Atari et al., 2017c), and consideration of cosmetic surgery (Swami et al., 2009; Chegeni and Atari, 2017). Individual differences therefore may co-vary with performance frequencies of mate retention behavior and consideration of cosmetic surgery. Women scoring higher (versus lower) on Machiavellianism, for example, may be more willing to manipulate their perceived mate value via cosmetic procedures as a means of Benefit-Provisioning mate retention. Future research investigating cosmetic surgery from within an evolutionary framework may benefit from examining the influence of personality dimensions and individual differences.

Finally, although the current findings demonstrated the existence of significant associations Between mate retention behaviors and consideration of cosmetic surgery, directionality cannot be determined as a result of our reliance on cross-sectional correlational data. For example, it is possible that the direction of causality may be reversed such that the proclivity to improve physical appearance may influence report of different mate retention behaviors. Furthermore, it is possible that a reciprocal relationship exists between consideration of cosmetic surgery and mate retention behaviors or that both variables develop as a result of another basic variable such as personality traits.Designing longitudinal studies in the future can provide insights into how undergoing cosmetic surgery actually affects romantic relationship outcomes. For example, it is currently unclear whether individuals’ performance frequency of mate retention behaviors changes following cosmetic procedures in women and men.

## Conclusion

Women, relative to men, are more likely to use appearance enhancement tactics as part of a broader strategy of Benefit-Provisioning mate retention to minimize the likelihood of partner infidelity (Buss, 1988). The current study examined whether consideration of cosmetic surgery was associated with performance frequencies of mate retention behaviors. The results reveal a positive association between consideration of cosmetic surgery and performance frequencies of Benefit-Provisioning mate retention for women, but not men. Thus, women may undergo cosmetic surgery to increase their perceived attractiveness as a means to retain their romantic partner.

## Ethics Statement

Participants were eligible to participate in this survey if they were at least 18 years of age and in a committed, heterosexual relationship. Interested and eligible participants gave consent. This study was carried out in accordance with the recommendations of the ethics committee of the Department of Psychology of University of Tehran. All subjects gave written informed consent in accordance with the Declaration of Helsinki.

## Author Contributions

All authors developed the study concept and contributed to the study design. MA and RC collected the data. MA, NB, YS, and TS performed the data analyses. MA, NB, and RC drafted the manuscript. YS and TS provided critical revisions to the manuscript. All authors reviewed and approved the final version of the manuscript for submission.

## Conflict of Interest Statement

The authors declare that the research was conducted in the absence of any commercial or financial relationships that could be construed as a potential conflict of interest.
